# Improving Access to Developmental Assessments Before School: Evaluation of Targeted ‘School Starter Blitz’ Clinics in Metropolitan Sydney

**DOI:** 10.1177/21501319251394543

**Published:** 2025-11-25

**Authors:** Laura Meyers, Pankaj Garg, Romy Hurwitz, Sinthu Vivekanandarajah, Lydia So, Suky Yim

**Affiliations:** 1 Liverpool Hospital, NSW, Australia; 2 University of New South Wales, Sydney, NSW, Australia; 3 South western Sydney Local Health District, NSW, Australia

**Keywords:** developmental assessment, waiting times, children, service redesign, CALD

## Abstract

**Background:**

Timely developmental assessment is essential for children with neurodevelopmental concerns, particularly before starting school. In public health systems, long wait times for multidisciplinary assessments disproportionately affect vulnerable populations, especially those from culturally and linguistically diverse (CALD) backgrounds. To address this, the South Western Sydney (SWS) Local Health District introduced ‘School Starter Blitz’ clinics—targeted initiatives prioritising school-entry-age children for diagnostic assessment.

**Methods:**

This retrospective cohort study analysed data from October 2022 to September 2024 for children aged 1 to 16 years referred to the Child Development Assessment Service (CDAS) with the objective of assessing impact of Blitz intervention on waiting times. Blitz clinics were held during 4 targeted months. Structured phone screening prioritised children nearing school age, particularly those without previous diagnoses. Demographic, clinical, and service-use data were analysed using descriptive and inferential statistics.

**Results:**

Of 1957 eligible children, 23.5% were assessed during Blitz months. These children were more likely to be younger (≤5.5 years), first-time patients, without prior diagnoses and classified as high priority at the time of referral triage (39% vs 22%, *P* < .001). Waiting times were significantly shorter reduced by 89 days on average (223 vs 312 days, *P* < .001) during blitz months. Regression analysis identified CALD background and children with diagnosis other than autism as additional factors associated with shorter wait times. Despite the Blitz months having generally shorter waiting times for various clinical factors, overall, there was no significant difference according to socioeconomic place of residence.

**Conclusion:**

The ‘School Starter Blitz’ effectively prioritised younger, first-time children from CALD backgrounds for developmental assessment. It represents a feasible and scalable service redesign that improves access and reduces delays in assessment. However, successful implementation requires investment in administrative support and staff satisfaction. The findings also underscore the persistent influence of the Inverse Care Law in public health access. Broader adoption of this model has the potential to enhance both equity and efficiency across public health systems.

## Introduction

Timely developmental assessments are crucial for children with suspected neurodevelopmental difficulties, yet waiting times remain unacceptably long across many health systems, including Australia.^1-3^ These delays have serious consequences, as early diagnosis enables timely intervention, supports children’s neurodevelopment in critical early years and reduces stress and uncertainty for families.^4^

Prolonged wait times in public developmental services are influenced by multiple structural and systemic factors. Chief among these are limited workforce capacity and chronic funding constraints that restrict the availability and reach of services.^5^ These systemic bottlenecks are compounded by inequities in access, and fragmentation of primary, secondary and tertiary systems for developmental surveillance and assessments.^6^ Research shows that factors like low socioeconomic status, limited maternal education, cultural and linguistic diversity (CALD) background, poor parental understanding of development and family vulnerabilities can delay recognition of developmental concerns and result in late referrals to assessment services.^5,7^ The Australian Bureau of Statistics enumerates a standard set of cultural and language indicators, such as country of birth of the person, main language other than English spoken at home, proficiency in spoken English, Indigenous status, ancestry, country of birth of parents, spoken languages at home, religious affiliation and year of arrival in Australia, to identify people of CALD backgrounds. Actively prioritising these groups in the assessment pathways are thus essential to avoid further delays in diagnosis and support.

Neurodevelopmental concerns frequently become apparent after children begin formal schooling, where classroom demands on attention, language, social interaction and adaptive behaviour reveal previously unnoticed difficulties even in children with risk factors such as prematurity.^8,9^ This has led to increased recognition of the importance of conducting standardised developmental assessments before school entry, ensuring children receive appropriate support from the start of their educational journey.^7^

Despite this increasing recognition, data suggest that many children are not assessed in time. A study conducted between 2019 and 2022 at a child developmental assessment unit in Sydney reported that fewer than 50% of children were assessed before the age of five.^7^ National Australian data also reflect this issue. The Australian Early Development Index (AEDI) found that although 4.4% of children had diagnosed special needs, teachers reported concerns in multiple developmental domains for 14.6%, mainly involving speech, emotional regulation and home environments.^10^ Alarmingly, this discrepancy appears to be worsening in certain regions in the most recently released AEDI Census data, indicating a growing mismatch between developmental needs and service availability. These findings reinforce the need for earlier identification and intervention and suggest that service redesign may be required to address gaps in access and identification of children with significant neurodevelopmental concerns.^11^

In response to long wait times and fragmented access, South Western Sydney’s (SWS) Child Developmental Assessment Service (CDAS) undertook a major service redesign in 2016. This involved implementing a standardised intake and triage system (prioritisation process for children to be seen within 3, 6 or 12 months), supported by a centralised database, to streamline multidisciplinary assessment and prioritise children with complex developmental or psychosocial needs.^12^ Referrals were accepted from within the district from paediatricians, allied health and schools with prioritisation guided by urgency, particularly for children with limited prior assessment, complex presentations or those approaching school age. This redesign marked a shift from reactive service delivery to proactive, needs-based planning (CDAS Model of Care, Supplemental File).

A key insight from the implementation of the CDAS re-design was the need for targeted support for children nearing school entry. In response, the ‘School Starter Blitz’ clinics were introduced, an innovation focused on timely assessment for this critical group.

This paper is part of the broader evaluation of the CDAS service redesign, specifically assessing the impact of the School Starter Blitz clinics. The objectives of the present study were to examine whether these clinics effectively reduced wait times and compares the demographic and clinical profiles of children seen during the Blitz periods with those seen in other months. The findings aim to guide future service planning, resource allocation and strategies to enhance developmental care access for vulnerable populations in the region.

## Methods

### Study Setting

The study was conducted in the South Wsetern Sydney metropolitan region, one of the most culturally and linguistically diverse districts in Sydney.^13^ Over half the residents speak a language other than English, and the region has more children under 14 than the New South Wales state average. Common languages include Arabic, Vietnamese, Cantonese, Mandarin, Spanish, Hindi, Bengali, Italian, Turkish and Greek. It also has high rates of resettled refugees and a greater prevalence of neurodevelopmental concerns and disabilities than the state average.^14^

## Blitz Intervention

### School Starter Blitz Clinic Screening Process

The South Western Sydney’s Child Development Assessment Service created a screening and booking process for children starting kindergarten the following year. This included all consecutive children who were referred to the service and waiting for developmental assessments. The eligible age for starting school varies depending on the local Department of Education rules. Children must start school in the year they turn 6, but they can begin earlier if they turn 5 by July of that year. The screening process involved contacting parents by phone and asking structured questions to assess the child’s readiness for school. The screening script included questions about school registration (type and placement), any developmental or psychometric assessments since the initial referral was made, new diagnoses (eg, developmental delay, autism), use of early intervention services, regular paediatrician involvement and recent recommendations. The family’s capacity to attend on short notice, flexibility to travel to alternative Community Health Centre sites beyond the nearest referral location and need for Interpreter was also ascertained. Parental consent was also required to confirm willingness to attend during the School Starter Blitz clinic month. This process aimed to prioritise timely assessment and support for children approaching school entry.

Based on the responses, 2 outcomes were possible: (1) discharge with parental consent if the child had a confirmed diagnosis with appropriate supports, and set up for school, or if the family declined the assessment, with notification to the original referrer and/or the child’s Paediatrician; or (2) booking an appointment during the designated ‘School Starter Blitz’ clinic if the child was deemed to be suitable.

### Inclusion Criteria

Children in both groups: the Blitz and non-Blitz cohorts were on CDAS waiting lists after being referred by Paediatricians or allied health specialists. The analysis included the Blitz group who were all the children that underwent standardised developmental assessments within the Blitz periods that had been prioritised and selected by the aforementioned process. The non-Blitz group were all other consecutive children up to 16 years that were assessed in all the other (non-Blitz) months. More than 90% of children seen by our service (Blitz and non-Blitz) are less than 7 years of age).^15^ We did not limit the age group to only preschool years to allow for comparison between Blitz and non-Blitz months (as older children are typically seen during the non-Blitz months). Our goal was to ascertain if we prioritised the right target groups during the Blitz months as per our priori hypothesis. This included children who may be more than 5 years but with no prior assessments or who may have missed out on services, and need to be prioritised and assessed.

### Blitz Clinic Process

The Blitz intervention consisted of firstly identifying children who require prioritisation for a developmental assessment, before undertaking comprehensive developmental evaluations using standardised tools. A detailed history is taken, observations are conducted, and relevant information is gathered from the families and other sources by a psychologist or developmental paediatrician. Common tools used during the Blitz months were Griffiths III Scales (27.9%, 326/1166), Vineland III/Adaptive Behaviour Assessment System, Third Edition (26.2%, 22/84) and the Autism Diagnostic Observation Schedule (10.6%, 21/199), cognitive tests (15.3%, 31/203). Comprehensive feedback is provided to families either during the same visit or at a follow-up appointment.

Typically, appointments last around 90 to 120 min. Feedback is also provided in the form of a detailed written report summarising all aspects of the assessment. This report is subsequently shared with early childhood intervention providers, preschools or public-school systems to ensure timely and appropriate intervention and educational support for the child.

### Data Collection

The study design followed principles of a retrospective nested case-control where children seen in Non-Blitz months acted as controls for children seen in the Blitz months.^16^ Data were extracted from the SWS CDAS database over 2 years (October 2022-September 2024), covering the ‘School Starter Blitz clinics’ held in November 2022, June 2023, November 2023, and May 2024. These clinics strategically reallocated CDAS resources to prioritise children, reflecting a flexible, responsive service model.^15^

Demographic variables that were collated included age, gender, suburb, country of birth, cultural background, CALD and Indigenous status, child protection involvement, out-of-home care, refugee status, referral details, priority, diagnosis, level of delays, comorbidities and waiting times. All data were de-identified.

### Data Cleaning

**Figure 1.**
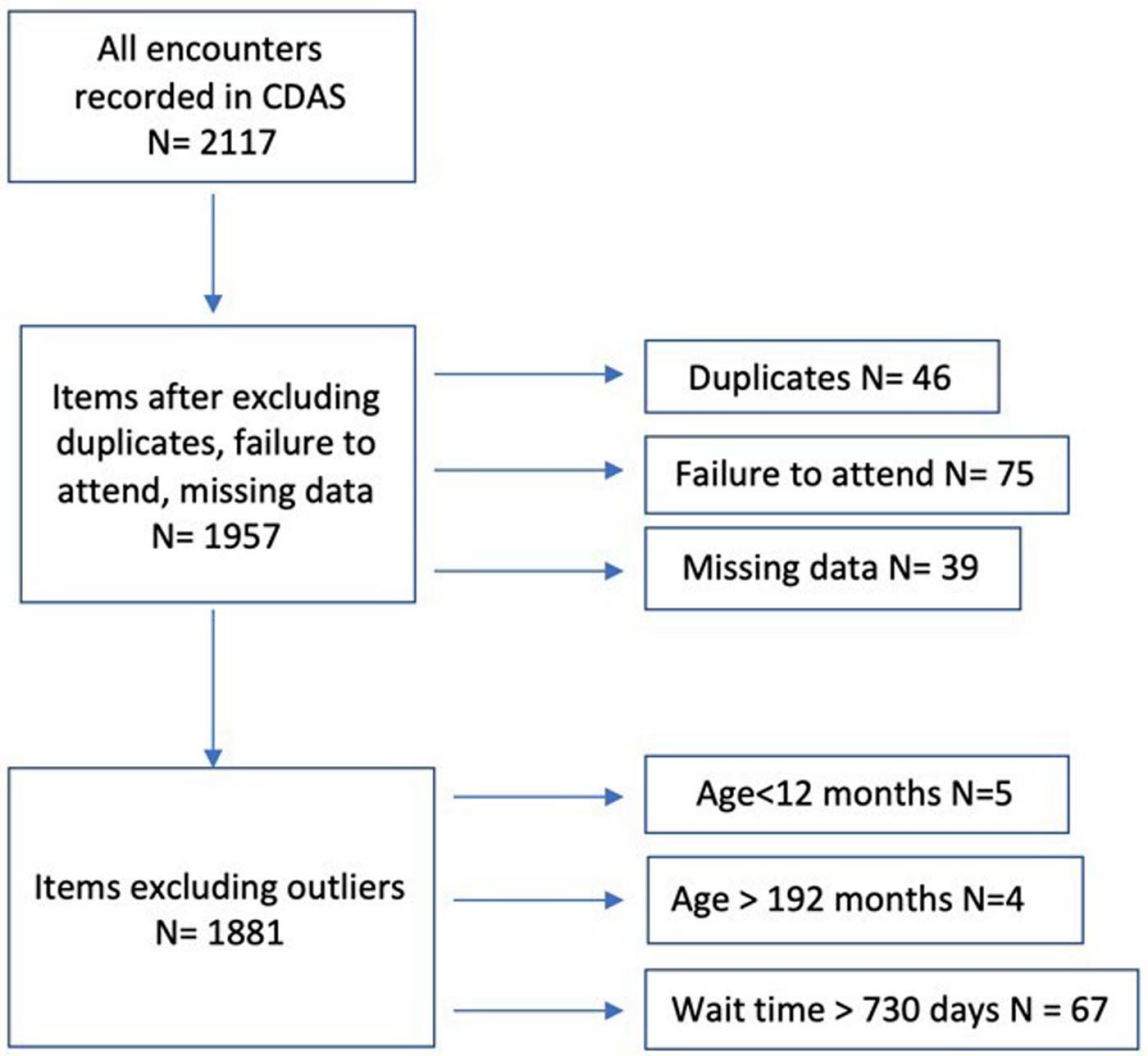
Data cleaning process indicating exclusions.

Of the initial 2117 encounters, records were excluded if incomplete, duplicated, or marked as ‘failure to attend’. Children waiting over 730 days were treated as outliers and were deemed appropriate for separate analysis to understand the factors leading to prolonged delays. We noted from a separate analysis of this cohort that most common factors of such prolonged wait times were family location changes, inability to contact families and administrative in nature such as wrong date of referral been recorded so this group was considered an outlier. This group was also less than 5% of the cohort and did not affect overall analysis presented in current report. Additional 9 children were age outliers, those under 12 months or over 192 months (16 years) were also excluded, as these age groups are typically managed by other community paediatric services rather than the CDAS Service (Figure 1).

### Data Analysis

Descriptive statistics summarised demographic characteristics, diagnostic categories and referral information. Frequencies and percentages described categorical variables, while geometric means, medians and interquartile ranges were used for continuous variables based on data distribution. Comparative analyses assessed differences between ‘School Starter Blitz’ and non-Blitz groups using one- and two-way ANOVA for continuous variables and Chi-square tests for categorical variables. Demographic and child-related variables were analysed to identify factors influencing waiting times. Histograms assessed waiting time distribution; non-normal data were log-transformed. Geometric means with 95% confidence intervals were reported to reduce skewness, consistent with prior health service waiting time analyses.^17^ This provided a more accurate measure of central tendency by reducing the impact of extreme values.^18^

Socioeconomic status was assessed using the Australian Bureau of Statistics, Socio-Economic Indexes for Areas **(**SEIFA), Index of Relative Socio-Economic Disadvantage (IRSD), with scores and deciles (1 = most disadvantaged, 10 = least) extracted at the level of post code.^19^ Associations between socioeconomic disadvantage and service waiting times were examined using appropriate statistical tests. Missing data was analysed for patterns using Multiple Imputation analysis method using random number generator and appropriate statistical methods.^20^ Multiple regression was performed for variables of clinical significance by entering variables using enter option.

A *P*-value of less than .05 was considered statistically significant. All analyses were performed using MedCalc Statistical Software version 23.0.8 (MedCalc Software Ltd, Ostend, Belgium; https://www.medcalc.org; 2024) except multiple imputation analysis which was done using IBM SPSS Statistics for Windows, version 30 (IBM Corp., Armonk, N.Y., USA).

### Ethics Approval

Ethics approval was granted by the SWSLHD Quality Improvement Committee (PCH01/2024/08). Consent was waived as all data were de-identified, and no patient contact occurred.

## Results

### Demographics

Data from 1957 patients were analysed for baseline demographics, with 1881 children included in the waiting time analysis (Figure 1). The cohort had a median age of 57 months (IQR 48-65), with 71% boys and almost 3-quarter were under 65 months. Most referrals came from paediatricians (76%), and almost 3-quarters were new to the service. Half of the children were from culturally and linguistically diverse (CALD) backgrounds, though the majority did not require interpreters and about 10% had additional vulnerability factors such as out-of-home care, FACS involvement, refugee status, or Aboriginal/Torres Strait Islander background. Moderate developmental delay was the most common level of delays observed in almost 40%.

### School Starter Blitz Clinic versus non-blitz Clinic Cohorts

Table 1 summarises demographics of School Starter Blitz and non-Blitz groups. About 23.5% were seen during Blitz months. Blitz patients were more likely ≤ 65 months (81% vs 74%, *P* = .001) and new patients (*P* < .001). Both groups’ demographics were similar, matching the overall cohort except those children born overseas (*P* < .0001) and referral sources other than allied health and paediatricians such as schools (*P* = .002) was more common in children seen during Blitz clinic months.

**Table 1.** Comparison of Characteristics Between Blitz and Non-Blitz Groups (n = 1957).

Characteristic	Blitz n (%)	95% CI Blitz	Non-Blitz n (%)	95% CI Non-Blitz	Chi-square (χ²)	*P*-value
Age at presentation (months)						
≤65 months (n = 457)	370 (81.0%)	77.2%-84.8%	1106 (74.1%)	72.0%-76.3%	10.6	.001^a^
>65 months (n = 474)	87 (19.0%)	15.2%-22.8%	387 (25.9%)	23.7%-28.0%		
Gender (n = 1956)						
Female	133 (29.1%)	24.9%-33.5%	423 (28.4%)	26.4%-30.7%	1.3	.53
Male	323 (70.7%)	69.0%-73.6%	1064 (71.3%)	66.5%-74.9%		
Unknown	1 (0.2%)	0.0%-0.5%	5 (0.3%)	0.0%-0.7%		
Years, seen (n = 1957)						
Oct 2022-Dec 2022 (n = 260)	131 (50.4%)	44.5%-56.3%	129 (49.6%)	43.7%-55.5%	97.5	<.0001^a^
Jan 2023-Dec 2023 (n = 1027)	232 (22.6%)	20.0%-25.2%	795 (77.4%)	74.8%-80.0%		
Jan 2024-Sep 2024 (n = 670)	97 (14.5%)	11.7%-17.3%	573 (85.5%)	82.7%-88.3%		
Referral source (n = 1956)						
Paediatrician (n = 1481)	347 (23.4%)	21.2%-25.6%	1134 (76.6%)	74.4%-78.8%	14.6	.002^a^
Allied Health (n = 443)	105 (23.7%)	19.7%-27.7%	338 (76.3%)	72.3%-80.3%		
Other (n = 27)	20 (74.1%)	56.2%-92.0%	7 (25.9%)	8.0%-43.8%		
Missing data (n = 5)	1 (20.0%)	0.0%-54.0%	4 (80.0%)	46.0%-100.0%		
Country of birth (n = 1949)						
Australia (n = 1838)	432 (23.5%)	21.5%-25.5%	1432 (76.5%)	74.5%-78.5%	90.5	<.0001^a^
Overseas (n = 111)	90 (81.1%)	73.3%-89.0%	21 (18.9%)	11.0%-26.7%		
Cultural background (n = 1949)						
European (n = 589)	129 (21.9%)	18.5%-25.3%	460 (78.1%)	74.7%-81.5%	10.33	.41
Pacific Islander (n = 49)	12 (24.5%)	11.9%-37.1%	37 (75.5%)	62.9%-88.1%		
Indian subcontinent (n = 137)	34 (24.8%)	17.7%-31.9%	103 (75.2%)	68.1%-82.3%		
Chinese (n = 41)	11 (26.8%)	12.5%-41.1%	30 (73.2%)	58.9%-87.5%		
Vietnamese (n = 102)	26 (25.5%)	17.0%-33.9%	76 (74.5%)	66.1%-82.9%		
Asian Other (n = 206)	58 (28.2%)	21.8%-34.6%	148 (71.8%)	65.4%-78.2%		
Arabic/Middle East (n = 256)	60 (23.4%)	18.1%-28.7%	196 (76.6%)	71.3%-81.9%		
Mixed ancestry (n = 156)	36 (23.1%)	16.3%-29.9%	120 (76.9%)	70.1%-83.7%		
Other (n = 188)	49 (26.1%)	19.8%-32.4%	139 (73.9%)	67.6%-80.2%		
Unknown (n = 225)	47 (20.9%)	15.3%-26.5%	178 (79.1%)	73.5%-84.7%		
CALD status (n = 1950)						
CALD (n = 980)	243 (56.8%)	52.8%-60.8%	737 (52.7%)	49.8%-55.6%	4.16	.12
Not CALD (n = 846)	185 (43.2%)	39.2%-47.2%	661 (47.3%)	44.4%-50.2%		
Unknown (n = 124)	29 (23.4%)	16.0%-30.8%	95 (76.6%)	69.2%-84.0%		
Interpreter (n = 1949)						
Interpreter used (n = 460)	52 (11.3%)	8.5%-14.7%	129 (8.7%)	7.3%-10.2%	3.1	.07
No Interpreter (n = 1489)	408 (%)	85.3%-91.5%	1360 (91.3%)	89.8%-92.7%		
Any vulnerable factors (n = 1947)						
Vulnerable (n = 199)	49 (10.7%)	7.9%-13.5%	150 (10.1%)	8.5%-11.7%	0.22	.63
No vulnerable (n = 1748)	408 (89.3%)	86.5%-92.1%	1340 (89.9%)	88.3%-91.5%		
Vulnerability by CALD (n = 1823)						
CALD with vulnerable (n = 70)	21 (30.0%)	18.7%-41.3%	49 (70.0%)	58.7%-81.3%	15.8	<.0001^a^
Non-CALD vulnerable (n = 115)	75 (65.2%)	56.2%-74.2%	40 (34.8%)	25.8%-43.8%		
Appointment type (n = 1950)						
New (n = 1367)	361 (84.3%)	81.0%-87.6%	1006 (72.6%)	70.4%-74.8%	15.8	<.0001^a^
Review (n = 447)	67 (15.7%)	12.4%-19.0%	380 (27.4%)	25.2%-29.6%		
Level of Delay (n = 1586)^b^						
Average (n = 270)	55 (13.8%)	10.3%-17.3%	215 (18.1%)	15.9%-20.3%	7.75	.051
Mild (n = 550)	145 (36.7%)	32.0%-41.4%	405 (33.9%)	30.8%-36.9%		
Moderate (n = 615)	159 (40.8%)	36.2%-45.4%	456 (38.2%)	34.9%-41.5%		
Severe (n = 151)	35 (8.7%)	5.9%-11.5%	116 (9.7%)	8.0%-11.4%		
Diagnosis (n = 1957)						
Autism spectrum disorders (n = 946)	226 (23.9%)	21.2%-26.6%	720 (76.1%)	73.4%-78.8%	0.35	.55
Other Neurodevelopmental (n = 1011)	255 (25.2%)	22.5%-27.9%	756 (74.8%)	72.1%-77.5%		
Co-morbidities (n = 1957)						
Chronic health issues (n = 196)	52 (26.5%)	20.3%-32.7%	144 (73.5%)	67.3%-79.7%	3.9	.27
Known genetic condition (n = 50)	9 (18.0%)	7.1%-28.9%	41 (82.0%)	71.1%-92.9%		
Neurological/Mental/Behavioural (n = 137)	40 (29.2%)	21.4%-37.0%	97 (70.8%)	63.0%-78.6%		
No comorbidities recorded (n = 1574)	346 (22.0%)	19.8%-24.2%	1228 (78.0%)	75.8%-80.2%		

a Statistically significant, *P* < .05.

b Level of delays were estimated clinically using quartile charts of Griffiths III on developmental ages, and calculated DQs, <50 was moderate and if ≤25 to 30 were considered severe, Average skills kids often had High functioning autism or other neurodevelopmental issues as Attention deficit hyperactive disorder, other behavioural problems, complex childhood trauma etc.

**Figure 2.**
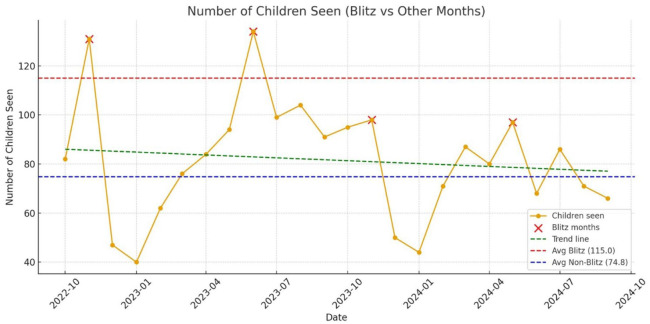
Number of children seen in Blitz months versus non-Blitz-months.

Figure 2 shows more children seen during Blitz months (Nov 2022, Jun 2023, Nov 2023, May 2024) than non-Blitz months.

### Differences in Demographic Variables, Waiting Times and Prioritisation Measures

Significant differences were found in the triage priority and appointment priority between groups. More children in the Blitz group were assigned Priority 1 at triage (38.6%) and received Priority 1 appointments (28.5%) than the non-Blitz group (22.4% and 14.4%, respectively). About two-thirds of children in both groups were seen within or earlier than their allocated triage time frame (64.0% Blitz, 66.2% non-Blitz, *P* = .43). Similarly, there was no difference in the proportion of children seen longer than their priority allocation time frame (36% Blitz, 33.8% non-Blitz).

The Geometric mean waiting time for the School Starter Blitz group was significantly lower (223 days) than that for the non-Blitz group (312 days), as shown in Table 2. Statistically significant reductions in waiting times were also observed among children aged ≤65 months during Blitz months compared to non-Blitz months (303.1 days). When examining waiting times across different SEIFA indices quartiles within the cohort, overall, no statistically significant differences were found although for each quartiles waiting times were shorter for Blitz group (Table 2).

**Table 2.** Mean Waiting Times With 95% Confidence Intervals.

Variable	Blitz group Mean waiting time (95% CI)	Non-Blitz Group Mean waiting times (95% CI)	*P*-value
Overall Geometric means waiting times in Blitz vs Non-Blitz Cohorts	223.9 (204.6-242.7)	312.2 (299.7-325.3)	<.0001*
Age at presentation (months)			.049*
≤65 months (n = 457)	303.1 (264.8-341.3)	365.25 (343.1-387.3)	<.001*
>65 months (n = 474)	420.4 (341.5-499)	580.8 (543.3-618.2)	.001*
Gender (n = 1956)			.05*
Female	363.5 (299.1-428.1)	407.1 (370-243.3)	.009*
Male	311.2 (269.7-352)	418.2 (395.2-441)	.003*
Years, seen (n = 1957)			.6
Oct 2022-Dec 2022 (n = 260)	384.8 (318.8-450)	461.5 (394-528)	.05*
Jan 2023-Dec 2023 (n = 1027)	301.3 (251.7-350.9)	401.6 (374-428)	.037*
Jan 2024-Sep 2024 (n = 670)	302 (226.3-379)	438.7 (407-470)	<.001
Referral source (n = 1956)			.06
Paediatrician (n = 1481)	326 (286-367)	426 (404-448)	.56
Allied Health (n = 443)	304.2 (230.6-377)	402 (362-443)	.41
Other (n = 27)	301 (129-473)	602 (318-886)	.002*
Country of birth (n = 1949)			.05
Australia (n = 1838)	410.18 (249-570)	499 (420-578)	.9
Overseas (n = 111)	321.1 (285-357)	415.9 (395-436)	.06
CALD status (n = 1950)			.97
CALD (n = 980)	287.4 (231-343)	387 (357.4-416)	<.001*
Not CALD (n = 846)	350.7 (301-399.5)	451.5 (423.5-479.6)	.003*
Interpreter (n = 1949)			.046*
Interpreter used (n = 460)	315 (278-353)	418 (398-438)	.12
No Interpreter (n = 1489)	403 (299.8-507)	433 (367-499)	.26
Any vulnerable factors (n = 1947)			.8
Vulnerable (n = 199)	326 (218-435)	432 (371-493)	.003*
No vulnerable (n = 1748)	325 (287.9-362.5)	419.7 (399-440)	.8
Vulnerability by CALD (n = 1823)			.8
CALD with vulnerable (n = 70)	305 (157-453)	379.8 (299-460)	.46
Non-CALD vulnerable (n = 115)	326.6 (290.2-362.9)	423.5 (403.4-443.7)	.05*
Appointment type (n = 1950)			.94
New (n = 1367)	288.8 (250-327)	541 (452-630)	<.001*
Review (n = 447)	358 (335.1-381)	601 (564-638)	.02*
Level of delay (n = 1586)			.98
Average (n = 270)	253 (147-360)	496 (442-549)	<.001*
Mild (n = 550)	299.8 (233-365)	432 (392.8-471.3)	.02*
Moderate (n = 615)	356 (293-419)	391 (354-428)	.5
Severe (n = 151)	315 (181-448)	439 (366-513)	.02*
Diagnosis (n = 1957)			.9
Autism spectrum disorders (n = 946)	381.1 (350.6-411)	443.6 (417-470)	.003*
Other Neurodevelopmental (n = 1011)	293 (242.7-344.6)	351.2 (303.8-398.6)	<.001*
SEIFA Indices Quartile			.6
1	352 (282-422)	427 (380-468)	<.001*
2	329 (258-400)	435 (393-477)	<.001*
3	281.8 (205-358)	436 (392-480)	<.001*
4	291 (212-375)	387 (345-428)	<.001*

* Statistically significant *P* < .05.

Mean waiting times also differed across other child-related variables between Blitz and non-Blitz months. Children with no confirmed diagnosis but significant developmental concerns waited mean of 272.8 days (95% CI 215.1-330.6) during Blitz versus 325.9 days (95% CI 290.7-361.2) in non-Blitz months (*P* = .13). While this difference was not statistically significant, this was noted to be clinically meaningful. Those with non-autism diagnoses had reduced waiting times from means of 351.2 (95% CI303.8-398.6) to 293 (242.7-344.6) days (*P* < .001). New patients also waited less in Blitz months 288.8 (95% CI 250-327) vs 351.2 (95% CI 303.8-398.6), *P* < .001, highlighting potential benefits of the Blitz model in reducing wait times.

A multiple regression analysis confirmed that children seen during Blitz months, CALD group, those seen for the first time by the service (new patients), children with diagnosis other than autism spectrum disorders and younger children had shorter waiting times (Table 3).

**Table 3.** Multiple Regression Analysis of Factors Affecting Waiting Times.*

Independent variable	Coefficient	Standard error	95% CI	*t*	*P*-value
Age > 65 months vs ≤ 65 months	141.2641	25.1035	92.0 to 190.5	5.6273	<.0001^a^
Blitz vs Non-blitz	−71.0498	21.3320	−112.8 to −29.2	−3.3307	.0009^a^
CALD vs non-CALD	−51.0402	20.6319	−91.51 to 10.56	−2.4738	.0135^a^
Interpreter Used	−20.0720	32.6632	−84.15 to 44.01	−0.6145	.5390
Level of developmental delays	3.2338	11.4602	−19.24 to 25.71	0.2822	.7779
New versus Review	−223.5215	23.4034	−269.43 to −177.61	−9.5508	<.0001^a^
Primary Diagnosis Autism versus others	40.4064	19.0329	3.06 to 77.74	2.1230	.0339^a^
Vulnerabilities with CALD vs Non-CALD with vulnerabilities	6.9094	38.5376	−68.69 to 82.50	0.1793	.8577

a Statistically significant.

* Enter option with variables retained if *P*< .05.

## Discussion

This study evaluated the impact of the ‘School Starter Blitz’ clinics on reducing waiting times for children requiring developmental assessments in a culturally diverse and socially complex metropolitan region in Australia. Our findings suggest that this targeted intervention improved timeliness of access for younger children approaching school entry from CALD backgrounds without compromising diagnostic thresholds or other vulnerabilities.

## Comparison With Previous Work

This study builds upon our earlier work undertaken in 2024, which analysed patterns of wait times, prioritisation and service access in relation to social vulnerability markers in Southwestern Sydney.^13^ That study reported a median wait time of 302.5 days, with only 42.6% seen within their triage timeframe.

In contrast, the current study found shorter wait times during School Starter Blitz periods (223.3 vs 310.9 days). Our overall waiting times did not increase compared to previous years though for all most variables Blitz months had shorter waiting times (Table 2). In the Blitz cohort, more children were triaged and seen as Priority 1, indicating improved alignment between triage and service delivery. These findings align with the global emphasis on early identification through structured developmental service models.^21^ Similarly, multi-strategy interventions such as rapid-access clinics and cross-sector developmental hubs in the United Kingdom have demonstrated improved diagnostic timeliness without reducing access for vulnerable populations.^22,23^

## Interpretation of Findings

The Blitz model increased the number of children seen and reduced wait times, particularly for those aged younger children ≤65 months and for new patients. The standardised phone-screening process also facilitated links to early interventions. Those from CALD groups experienced shorter delays. Some evidence also suggested prioritisation of vulnerable non-CALD children, though this finding lost significance in regression analyses, likely due to sample size limitations in that group.

No significant differences were observed across SEIFA socioeconomic indices, in contrast with prior Australian studies that reported delayed access among lower SES groups. This suggests the inverse care law was still present, with children from advantageous SEIFA deciles experiencing shorter wait times.^24,25^ Clinicians may have counterbalanced some systemic inequities during the referral triage process, as the CDAS service model prioritises age at school entry, lack of prior assessments and prioritisation based on information available on social vulnerabilities. However, the absence of individual-level socioeconomic variables (eg, income, maternal education) limits the interpretation on other social factors, as SEIFA only reflects an area-level disadvantage.

Interpreter use was higher during Blitz months, though statistical significance was not reached due to small sample sizes. Regression analysis nonetheless demonstrated clear prioritisation of CALD families, who are often at higher risk of service access barriers.

Reduced waiting times were also observed for children without prior diagnoses, and those with non-autism neurodevelopmental concerns. This supports the model’s transdiagnostic approach, addressing developmental needs beyond autism and promoting comprehensive early identification and support.^8^ Variability in the number of children seen (Figure 2) during Blitz months highlights ongoing resource and administrative challenges.^4^

Although the Blitz intervention has proven effective in reducing service backlogs of reducing waiting times, it is not currently feasible as a routine strategy due to its intensive nature. The key limitations include the need for significant resource reallocation, risk of staff burnout and limited administrative capacity. These factors highlight the need further research into staff satisfaction and wellbeing associated with Blitz interventions.

## Implications

Targeted interventions such as School Starter Blitz clinics can improve timely access to developmental assessments by reducing wait times without excluding at-risk groups. These findings support the feasibility of system-level approaches that maintain diagnostic assessments while potentially enhancing equity.

For policymakers, the Blitz model illustrates how family-centred, time-limited-service innovations can be embedded within existing child development assessment service models. Integration into national child development strategies, supported by digital triage tools, workforce training and cross-sector collaboration, may advance equitable and timely access at scale.

## Limitations

Despite real-world data and strong sample size, limitations exist. The retrospective design risks incomplete data, though regression and multiple imputation mitigated this. Lack of individual-level socioeconomic data is key, especially as shorter waits correlated with higher SEIFA deciles. A contemporaneous control group was lacking, but the nested case-control design (non-Blitz months as controls) and prior historical comparison addressed this.^13^ Long-term developmental outcomes were not assessed, limiting conclusions on sustained benefits. Diagnostic accuracy was not evaluated, being beyond the scope of current work. Absence of qualitative and cost-effectiveness data limits contextual insight and policy relevance. Unmeasured confounders (eg, staffing, resources, administration) may have affected wait times. A critical area for future investigation is staff satisfaction and wellbeing in relation to Blitz interventions. Understanding the impact of such intensive models on staff morale, job satisfaction and retention is essential for evaluating their sustainability. Findings are from a single health district with unique characteristics and may not generalise to rural/international settings. The sample focused on children nearing school age; results may not apply to older children or those with complex diagnoses. Some other factors that may have introduced bias include families not providing consent for participation in Blitz intervention, and families with higher vulnerabilities failing to attend their assessment appointments.

## Future Directions

To improve access, efficiency, and equity, Blitz clinics could expand across months and regions, particularly underserved areas. Telehealth assessments may increase access for families with barriers to in-person visits. Integrating services with early education settings could enhance early identification.^26-28^

A digital referral system could streamline communication among care providers, reducing delays. Future studies should assess cost-effectiveness to support scalability, as similar rapid-access models have shown policy relevance in other contexts.^22^ Machine learning tools incorporating child/family factors could refine triage, optimising resource allocation for those with highest need.

## Conclusion

The School Starter Blitz shows that focused, time-limited interventions can reduce wait times while supporting maintaining equity and standardised diagnostic assessments. For sustained impact, it should be embedded in broader child health strategies and adapted across diverse systems. Future research should use mixed methods to assess outcomes, combining both quantitative outcomes with qualitative perspectives, cost-effectiveness and long-term impact on children. For policymakers, Blitz offers a scalable, family-centred model. Embedding such approaches in national strategies, supported by digital tools and cross-sector collaboration, can improve developmental care, school readiness and long-term outcomes.

## Supplemental Material

sj-docx-1-jpc-10.1177_21501319251394543 – Supplemental material for Improving Access to Developmental Assessments Before School: Evaluation of Targeted ‘School Starter Blitz’ Clinics in Metropolitan Sydney Supplemental material, sj-docx-1-jpc-10.1177_21501319251394543 for Improving Access to Developmental Assessments Before School: Evaluation of Targeted ‘School Starter Blitz’ Clinics in Metropolitan Sydney by Laura Meyers, Pankaj Garg, Romy Hurwitz, Sinthu Vivekanandarajah, Lydia So and Suky Yim in Journal of Primary Care & Community Health

sj-pdf-2-jpc-10.1177_21501319251394543 – Supplemental material for Improving Access to Developmental Assessments Before School: Evaluation of Targeted ‘School Starter Blitz’ Clinics in Metropolitan Sydney Supplemental material, sj-pdf-2-jpc-10.1177_21501319251394543 for Improving Access to Developmental Assessments Before School: Evaluation of Targeted ‘School Starter Blitz’ Clinics in Metropolitan Sydney by Laura Meyers, Pankaj Garg, Romy Hurwitz, Sinthu Vivekanandarajah, Lydia So and Suky Yim in Journal of Primary Care & Community Health
