# Supplementary material for: Improving Access to Developmental Assessments Before School: Evaluation of Targeted ‘School Starter Blitz’ Clinics in Metropolitan Sydney
Source: J Prim Care Community Health. 2025 Nov 25;16:21501319251394543. doi: 10.1177/21501319251394543 (PMC12657776; doi:10.1177/21501319251394543)
Supplement: sj-docx-1-jpc-10.1177_21501319251394543 – Supplemental material for Improving Access to Developmental Assessments Before School: Evaluation of Targeted ‘School Starter Blitz’ Clinics in Metropolitan Sydney [file sj-docx-1-jpc-10.1177_21501319251394543.docx]

| **STROBE Item** | **Paper Alignment / Evidence** |
| --- | --- |
| **Title & Abstract** | Observational study evaluating School Starter Blitz; abstract summarizes objectives, methods, results, conclusions. |
| **Introduction / Background** | Provides scientific rationale; clearly states objectives and hypotheses regarding wait times and demographic comparisons. |
| **Study design** | Retrospective nested case-control; clearly described in Methods. |
| **Setting** | South Western Sydney; culturally diverse, population characteristics provided. |
| **Participants / eligibility criteria** | Inclusion/exclusion criteria for Blitz and non-Blitz cohorts clearly defined; age and referral sources reported. |
| **Variables** | Demographics, CALD status, Indigenous status, vulnerabilities, diagnosis, comorbidities, SEIFA indices, waiting times. |
| **Data sources / measurement** | CDAS database;structured phone screening. |
| **Bias** | Attempted to reduce selection bias; missing data addressed with multiple imputation; nested control design. |
| **Study size** | Total n=1,957; exclusions and outliers documented (Figure 1). |
| **Quantitative variables** | Waiting times, age, SEIFA deciles, diagnosis, priority levels, new vs. review appointments. |
| **Statistical methods** | Descriptive stats, ANOVA, Chi-square, regression, log-transformed skewed data; significance p<0.05. |
| **Participants flow** | Number included/excluded shown (Figure 1); demographic breakdown provided. |
| **Descriptive data** | Age, gender, CALD, vulnerabilities, referral source, level of delay, diagnosis, comorbidities. |
| **Outcome data** | Waiting times reported with 95% CI, geometric means, stratified by group, age, SES, diagnosis. |
| **Main results** | Blitz group had significantly reduced wait times; regression analysis identifies predictors (Table 3). |
| **Other analyses** | Differences by age, SEIFA quartiles, CALD status, vulnerabilities, appointment type. |
| **Discussion – Key results** | Interprets impact of Blitz clinics on wait times and service equity; compares to prior studies. |
| **Discussion – Limitations** | Retrospective design, missing individual-level SES, long-term outcomes not assessed, absence of qualitative/cost data. |
| **Discussion – Interpretation** | Explains findings in context of policy, early intervention, equity, and prior research. |
| **Discussion – Generalisability** | Addresses applicability to other regions and diverse populations. |
| **Ethics** | Approved by SWSLHD QI Committee; consent waived for de-identified data. |
| **Funding / Conflicts** | No funding and conflicts of Interest reported |
